# NAc-DBS selectively enhances memory updating without effect on retrieval

**DOI:** 10.1038/s41386-025-02132-0

**Published:** 2025-05-27

**Authors:** Andrés Pérez-Segura, Jorge Medina, Antonio Cerdán Cerdá, Cristina Sánchez-Ferri, Daniel Torres, Claudio R. Mirasso, Bryan Strange, Víctor M. Eguíluz, Lucas Lacasa, Santiago Canals

**Affiliations:** 1 Instituto de Neurociencias, Consejo Superior de Investigaciones Científicas & Universidad Miguel Hernández, Sant Joan d’Alacant, Spain; 2 Instituto de Física Interdisciplinar y Sistemas Complejos, IFISC (UIB-CSIC), Campus Universitat de les Illes Balears, Palma de Mallorca, Spain; 3 Laboratory for Clinical Neuroscience, Centre for Biomedical Technology, Universidad Politécnica de Madrid, Madrid, Spain; 4 Basque Centre for Climate Change (BC3), Scientific Campus of the University of the Basque Country, Leioa, Spain; 5 IKERBASQUE, Basque Foundation for Science, Bilbao, Spain

**Keywords:** Cognitive neuroscience, Learning and memory, Psychiatric disorders

## Abstract

Deep brain stimulation (DBS) has emerged as a widely used therapeutic option when pharmacological treatments prove ineffective or refractory for psychiatric patients. The nucleus accumbens (NAc) represents a frequently targeted site in DBS interventions due to its demonstrated safety profile and therapeutic efficacy in obsessive-compulsive disorder, major depression, and anorexia nervosa. However, limited mechanistic understanding hampers its broader clinical applicability. This study sought to delineate the distinct behavioural dimensions modulated by NAc-DBS, its impact on distinct facets of memory, and to elucidate the underlying brain-network mechanism of action. We developed a novel spatial navigation task for rats and employed a high-dimensional behavioural analysis complemented by fMRI to dissect the cognitive, behavioural and neurobiological effects of NAc-DBS. Active NAc-DBS in male rats produced a selective enhancement of long-term memory encoding without affecting memory recall or working memory. We found a small but statistically significant rewarding effect of NAc-DBS, with no detectable impact on motor or stress-related behaviours. Sustained neuronal activation in the NAc, septum, entorhinal and insular cortex demonstrated no desensitisation to chronic NAc-DBS, which triggered a functional reorganisation among dopaminergic-related structures. These findings suggest that NAc-DBS induces a functional reorganisation in the mesocorticolimbic system, potentially mimicking a dopaminergic novelty signal to enhance memory updating. This provides a mechanistic basis for the therapeutic use of NAc-DBS, particularly in improving cognitive flexibility in psychiatric disorders.

## Introduction

Neuromodulation has emerged as a valuable approach for psychiatric disorders resistant to pharmacological therapies [1, 2]. Among such techniques, deep brain stimulation (DBS) offers precise spatiotemporal control of neural activity. The nucleus accumbens (NAc), a basal forebrain structure central to reward, pleasure, and reinforcement learning, is a common DBS target in medication-refractory obsessive-compulsive disorder (OCD), major depression, or anorexia nervosa [3–5]. More broadly, DBS’s capacity to modulate neural activity highlights its potential to enhance cognitive function.

We recently reported that DBS in the NAc-medial septum improved mnemonic function in OCD patients [6], and other groups have similarly observed cognitive and memory benefits from NAc-DBS in humans [7, 8]. In animal models, several studies have implicated the NAc in the formation of hippocampal-dependent memories [9, 10], and we previously showed that disrupting the NAc function alters plasticity-related reorganisation of memory circuits [11]. Although the effects of NAc-DBS on OCD symptoms are not necessarily a direct consequence of memory enhancement, these findings suggest that clinical improvements may, at least in part, stem from enhanced memory updating, which could promote greater cognitive flexibility.

Despite extensive use, DBS’s mechanisms remain poorly understood. Many neurobiological processes and mechanisms likely contribute. Does DBS affect attention, memory encoding, retrieval, or other processes? And, mechanistically, how does the composition of the stimulated tissue or the electrical characteristics of the electrode and the stimulation protocols influence brain activation [12, 13]? Distinguishing direct from indirect modulation, determining whether orthodromic or antidromic signalling is engaged, the activation of *en passant* axons, and identifying synaptic plasticity changes are crucial. Animal models that allow circuit-level recordings and behavioural assessments are essential for clarifying these mechanisms.

The present study investigates how NAc-DBS influences short-term memory (STM), long-term memory (LTM) formation and recall simultaneously, using a novel behavioural paradigm partly adapted from previous work [14]. We employed high-dimensional behavioural analysis in rats and concurrent functional magnetic resonance imaging (fMRI) to measure DBS-induced changes in brain activity. Typically, animal memory studies rely on event counts, exploration times, or completion speeds, as in many loss- and gain-of-function experiments [15–18]. These measures often assess encoding, consolidation, or retrieval in isolation, overlooking confounds such as attention or locomotion. By dissecting the behavioural effects of DBS using a multi-dimensional analysis and uncovering the neural networks modulated in both the short and long term, we approach a more comprehensive understanding of how NAc-DBS influences memory processes, potentially guiding future neurotherapeutic interventions.

## Materials and methods

A total of 37 animals were used in four different experiments. Three behavioural experiments utilised 25 male Long Evans rats purchased from Janvier Labs (France), weighing around 250 g at the beginning of the experiments. They were paired and housed in a controlled environment with a room temperature of 21 ± 2 °C, following an inverted light cycle where lights were on from 20:00 to 8:00. This setup allowed behavioural procedures to be conducted during the rats’ dark cycle. Throughout the experiments, the rats had *ad libitum* access to food and water. 12 male Sprague-Dawley rats, purchased and housed under similar conditions, were used for the fMRI experiments.

The DBS protocol was designed to replicate clinical settings used in humans [1–3, 6, 19], employing biphasic pulses (cathodal phase first) at 130 Hz. Each pulse had a total duration of 120 μs and an amplitude of 250 μA (Fig. 3A). Stimulation was delivered bilaterally and continuously throughout behavioural task performance on designated days (see  Supplementary Information for surgical procedures and task descriptions). Trials with active and sham stimulation were randomly counterbalanced; in sham trials, animals were connected to the stimulator (STG-2004, Multi-Channel Systems, Germany) with the current set to zero.

For fMRI experiments, unilateral stimulation was used to enable detection of contralateral activations. Three stimulation paradigms were employed. To assess acute effects, a block design consisting of ten 8-s stimulation trains (ON periods) interleaved with 22-s OFF periods was used. The same protocol without OFF periods was applied to examine continuous stimulation effects. To assess DBS cessation effects—potentially revealing inhibitory rebound—BOLD responses were recorded during the transition from ON to OFF after 10 min of continuous stimulation (see Fig. 4B for schematics).

Detailed experimental procedures are provided in the Supplementary Information. All experiments were conducted in compliance with both Spanish (RD 53/2013) and European legislation (Directive 2010/63EU). The study was evaluated and approved by the CSIC Ethical Committee and authorised by local authorities.

## Results

To investigate the effect of DBS in the NAc on memory formation, we developed a behavioural navigation task inspired in the delayed-matching to place protocol of the Morris water maze [17]. This new task, and its detailed analysis, takes into account navigation trajectories, their directionality, persistence, biases and tendencies towards walls and central zones, as well as present and past target locations (see Supplementary Information). Collectively, these help to differentiate effects of DBS on memory encoding and retrieval in a single intervention.

### Spatial memory task

**Fig. 1:**
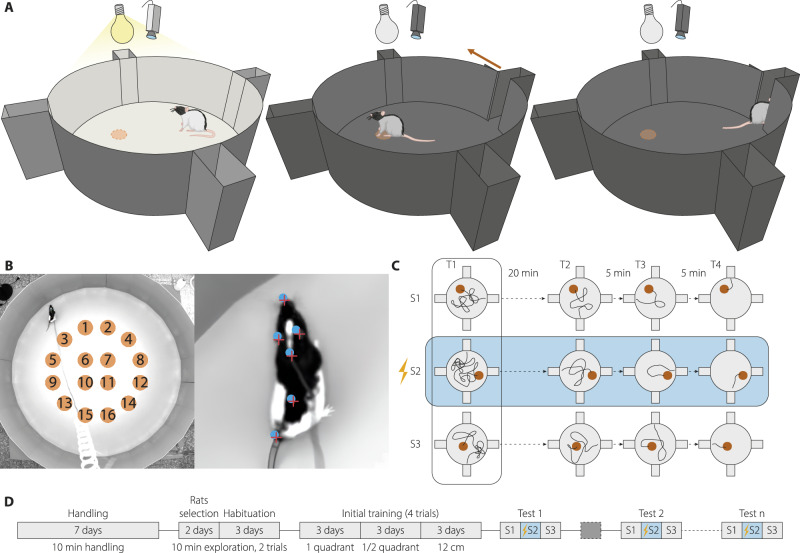
The spatial navigation task. **A** Representation of the circular arena (180 cm diameter) with the four entering boxes. The animal navigates in a brightly illuminated arena (400 lux) to find a computer-defined but invisible virtual platform (dotted orange circle). When the animal reaches the ‘platform’ the illumination dims and the exit door open allowing the animal to return to the entering box. **B** Image of the real maze from the top with the 16 possible virtual platform locations. In the right panel, a rat with the body parts used for tracking labelled (nose, left and right ears, shoulders, back and base of the tail). **C** Schematic of the three consecutive sessions (S1→S3), one per day, of a typical memory experiment, and the four trials performed each session for measuring and proper encoding the new ‘platform’ location. The location of the ‘platform’ is selected between the 16 preselected locations pseudo randomly to avoid repeating locations across days. Experimental manipulation (NAc-DBS) on the second day (S2, highlighted in blue) allows investigating memory retrieval, encoding and updating (see Supplementary Information). **D** Training schedule. After 7 days of handling and 5 of habituation, the animals completed 9 days of training to learn the task. Sequences of experimental sessions (S1→S3) were then intercalated with sessions in which the virtual platform does not change its location between days (dark grey box). Animals may repeat as many sequences of sessions as required.

In a large and brightly illuminated circular arena (diameter 180 cm), rats are trained to find a virtual platform with its successful localisation serving to dim the intense lights and open an escape door (Fig. 1A). The circular maze has 4 entrance/exit doors, and the entire operation is automated and guided in real time by video tracking. The virtual platform is a circular region of 12 cm diameter randomly placed in one of 16 predefined locations in the maze (Fig. 1B).

A specific feature of the design allows dissociation of memory formation and memory recall. The animals are extensively trained in this task for 15 sessions which are, importantly, structured in successive groups of 3 sessions called S1–S3 (Fig. 1C). After a period of habituation and training (Fig. 1D, Supplementary Information), an experimental session in 1 day consists of 4 trials (T1–T4) of up to 10 min in which the virtual platform remains in the same position (Fig. 1C). The location of the virtual platform changes the next day and is moved between the 16 locations across successive sessions. Thus, on T1, the animals may remember the location of the virtual platform of the previous session 1 day earlier; however, across trials T1 to T4, they will encode a new location and so form a new memory. This new location may be remembered on T1 of the next day. Accordingly, during the first 90 s of T1, the virtual platform is inactive to allow precise monitoring of the searching behaviour of the animals. By having successive groups of 3 sessions together, DBS could be applied on S2 to examine its impact on recall of memory formed on S1, its impact on STM during S2, and its impact on the formation of LTM measured on T1 of S3 (Fig. 1C). For offline behaviour quantification purposes, we defined a ROI with a diameter of 24 cm, centred on the virtual platform used in each session (see Supplementary Information).

**Fig. 2:**
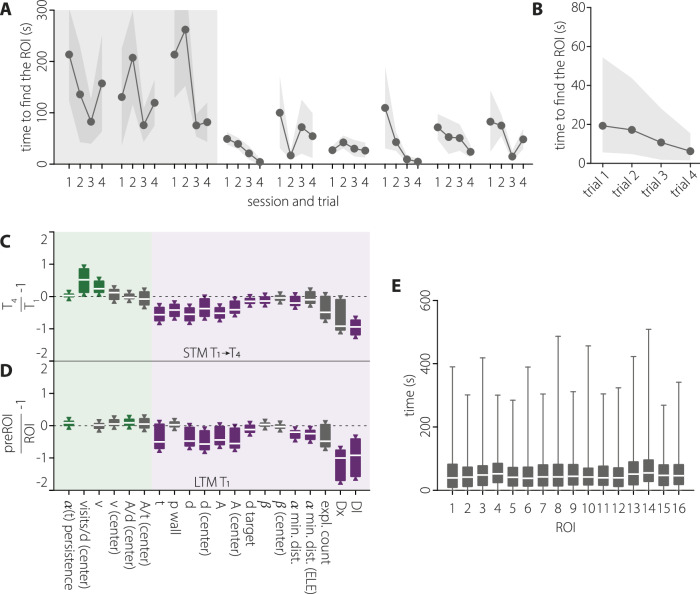
Memory evaluation in the navigation task. **A** Quantification of the time needed to find the ROI (an enlarged version of the virtual platform used to operate the maze, see Supplementary Information) of the rats across the last stage of the initial training (grey background) and the first six sessions, representing a typical performance. n = 6, mean ± SEM. **B** Quantification of the time needed to find the ROI in each trial performed after the completion of the initial training. 6 rats × 15 sessions, median ± interquartile range. **C** Quantitative evidence for STM. Equally tailed, 95% CI for the median ratio change between T1 and T4. Metrics on light green or light purple background are expected to adopt more positive or negative values, respectively, when the memory representation of the virtual platform (ROI) location improves. Green and purple-coloured boxplots indicate statistically significant differences at the 95% confidence level (one-tailed interval). **D** Same as in (**C**) for the quantitative evidence for LTM, measuring with respect to the ROI and the previous day’s ROI (preROI) in T1 across sessions. **E** Average time to find all possible ROI locations in all experiments. Median and min-to-max whiskers represented, n = 2910.

Once the initial training is completed (Figs. 1D, 2A), the duration for the animal to reach the ROI diminishes progressively across the four daily trials (repeated measures ANOVA Geisser-Greenhouse corrected, F(trial)_1.96, 147.7_ = 6, p = 0.003, Fig. 2B). This result underscores the animals’ understanding of task contingencies and allows quantification of STM within the session. The navigation of the animal on the following day (S2) towards the ROI location encountered on the previous day (S1) was, as noted, used to investigate LTM (Fig. 1C). The reason the searching time is long on T1 across multiple sessions is because the animals are remembering the location of the virtual platform of the previous day.

For each trial, we analysed the navigation patterns of the animals towards the target. A comprehensive set of 20 metrics delineating various aspects of navigation was extracted, which fell into 5 main categories: directionality towards target, wall navigation strategies, ballisticness, spatial navigation, and speed (see Table 1, Supplementary Information for details). Comparing animal trajectories between T1 and T4 of all sessions, unveiled statistically significant changes in 13 of these metrics after correcting for multiple comparisons, controlling for a false discovery rate (FDR) of 0.05. The joint evidence supports the acquisition of the new location into STM (Fig. 2C, Table S2). Notably, along the four trials, rats exhibited a more directed and less erratic navigation towards the target as indexed by the parameters *ß* and *α(t) persistence* (ballisticness), searched repeatedly in the correct area (*visits/d(center)*) and performed paths more directed to the virtual platform (*α min. dist*.), navigated closer to the target (*d target*) with less distance navigated, both in the central zone of the arena (*d(center)*) and overall (*d*), and needed to cover less area of the arena to find the target (*A* and *A(center)*, Fig. 2C). Probably as a consequence of the more efficient and directed exploration, animals found the target earlier (*t*, Fig. 2C). Moreover, the rats developed a navigation strategy characterised by circumnavigation around the walls to approach to the target ROI from a shorter distance to the wall, a behaviour quantified by the *Dl* metric (Fig. 2C). This strategy was accompanied by an increase in movement speed (*v*), increasing the overall proportion of time spent near the walls (*p wall*, Fig. 2C).

**Table 1:**
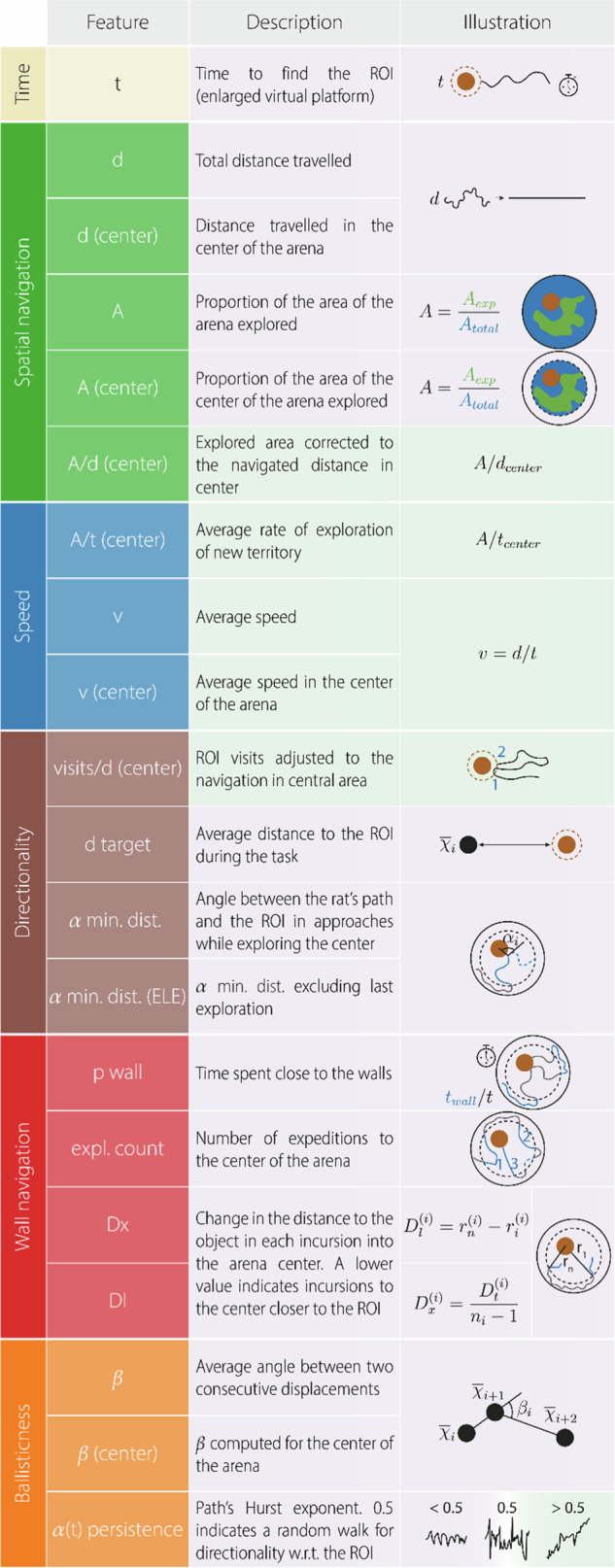
Behavioural features.

Representation of the metrics used to quantify STM and LTM. Each metric assesses different aspects of the rats’ navigation and is related to categories such as time (yellow), spatial navigation efficiency (green), speed (blue), directionality (brown), wall navigation strategies (red) and ballisticness (orange). Features on the purple or green background are expected, within this behavioural task, to decrease or increase, respectively, when navigation is guided by a more precise memory representation of the virtual platform location.

We then sought to determine whether any inherent biases could influence rats’ navigation towards specific regions of the arena, such as more centric vs. peripheral ROIs. We quantified the time required for rats to locate each potential ROI in every trial following the initial training phase. Our analysis revealed no significant differences in the time needed to find the various ROIs (Kruskal-Wallis test, χ^2^ = 23.59, p = 0.07, df = 15, Fig. 2E).

We next assessed the existence of LTM by analysing the navigation trajectories during the first trial (T1) on the following day (S2) towards the ROI that was active the previous day (S1). We compared the rat’s tendency to navigate towards the previous day’s ROI (S1) *versus* navigation directed to the current (still unknown) ROI (S2) or any other potential ROI location in the maze. It was observed that rats were more likely to reach first the S1’s ROI on T1 of S2 than the current ROI (time ratio to reach S1 or S2 ROIs in T1 of S2, P(*t*_S1ROI_ < *t*_S2ROI_) = 0.63, Table S1, Supplementary Information). This result could not be explained by random navigation, as indicated by the rejection of the null hypothesis stating that arrival is due to chance based on ROI proximity to the entering door (H_0_: *t*_closest_ < *t*_furthest_ rejected, Table S1, Supplementary Information), not being the closest ROIs the more likely to be reached first.

Importantly, rats also demonstrated in S2 significantly more directed navigation towards the S1 ROI (Fig. 2D, Table S2), taking less time to reach it (*t*, Fig. 2D), constraining their exploration trajectories to the area surrounding that ROI (*α min. Dist*., *α min. Dist. (ELE)*, *d target*, Fig. 2D) and tracing more ballistic trajectories (*α(t)* persistence, Fig. 2D) with less spatial redundancy (*A/d(center)*, Fig. 2D). Additionally, their exploration of the arena enroute to the previous session’s ROI was markedly more efficient, minimising the distance travelled, and the area covered before reaching the virtual platform (*d*, *d(center)*, *A*, *A(center)*, Fig. 2D). As for STM, rats exhibited in T1 the same circumnavigation strategy around the walls to approach the previous session’s target ROI from the shortest distance to the wall (*Dx*, *Dl*, Fig. 2D).

Information stored in LTM is progressively updated by the new information encountered in the arena over trials T1–T4, as searching first the previous ROI in T1 (significance level α = 0.05) was substituted by searching first at the current ROI in T4 (significance level α = 0.075).

### NAc-DBS experiments

**Fig. 3:**
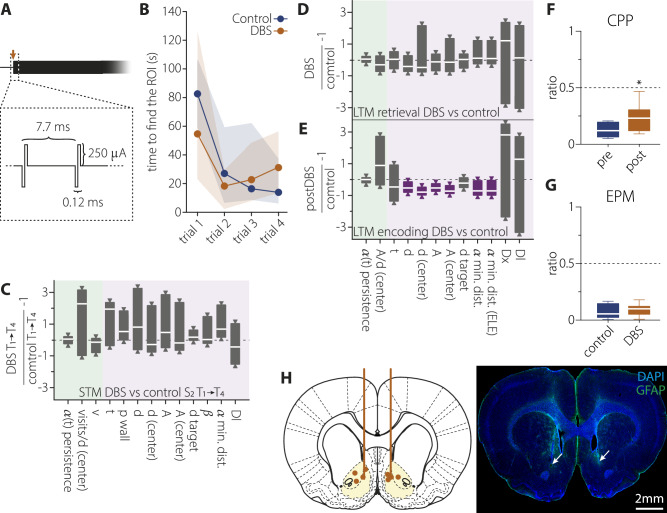
Effect of DBS in the NAc on memory performance. **A** Schematic representation of the DBS protocol, consisting of 250 μA biphasic pulses at 130 Hz delivered bilaterally and continuously during the 10 min of each trial of session S2 in selected experiments. **B** Quantification of the time to find the ROI in each trial in S2, in DBS-OFF (Control) and DBS-ON (DBS) conditions. 10 sessions for DBS-OFF and 8 sessions for DBS-ON, median ± interquartile range. **C** Effect of DBS on STM performance. Equally tailed 95% CI for the median ratio of metrics comparing DBS-OFF (Control) vs. DBS-ON (DBS) in T4. Metrics on green or purple background are expected to adopt more positive or negative values, respectively, when the memory representation of the platform location improves. Green and purple-coloured boxplots indicate statistically significant differences at the 95% confidence level (one-tailed interval). **D** Same as (**C**) for the effect of DBS on LTM retrieval, measuring at T1 of S2 the searching of the previous day’s ROI in sessions with DBS-OFF (Control) vs. DBS-ON (DBS). **E** Same as (**D**) for the effect of DBS on LTM encoding, measuring at T1 of S3 the searching of the ROI that was encoded the previous day during DBS-ON (postDBS) vs. DBS-OFF (Control). **F** Effect of DBS on CPP. Preference is measured as the ratio of the time spent in each chamber with respect the stimulated one. DBS is applied in the less-preferred chamber for each subject. Median and min-to-max whiskers represented, n = 7, *p < 0.05. **G** Effect of DBS on EPM. Exploration ratio between the open divided by closed arms, during DBS-OFF (Control) vs. DBS-ON (DBS) periods. Median and min-to-max whiskers represented, n = 7. **H** Schematic representation of the implanted electrodes with the tip of the electrodes marked by orange dots. Lower panel shows a representative example of a histological preparation. White arrows indicate the tip of the electrodes.

The previous experiments confirmed the existence of STM and LTM formation, both detectable within our experimental setup. Next, we examined the impact of NAc-DBS on memory formation over 4 successive series of three linked sessions (S1-3) in a within-subject manner, which increases the statistical power of the comparison. DBS was applied during the whole duration of each of the 4 trials of session S2, with BDS-ON and OFF sessions counterbalanced for each animal (see Fig. 3A).

Changes in STM and LTM induced by NAc-DBS are considered second-order effects with respect to the existence of STM and LTM (which would be the first-order effects). From a Bayesian perspective on metric design, we argue that the prior probability of a metric detecting a change in STM and LTM (the second-order effect) depends on its ability to detect the first-order effect. Consequently, the metrics designed to capture changes in STM and LTM in the NAc-DBS experiment are limited to the subset of the original 20 metrics that showed significant first-order effects. Metrics unable to detect such first-order effects cannot inform the study’s conclusions.

#### NAc-DBS effects on short-term memory

During STM formation, an analysis of arrival times to the ROI under both DBS-ON and DBS-OFF conditions, revealed a significant decrease between T1 and T4 trials in both groups, with neither stimulation effect or interaction between trials and conditions reaching significance (Mixed-effects model analysis F(trial)_1.5, 23.58_ = 6.28, p(trial) = 0.01, F(condition)_1, 16_ = 0.35, p(condition) = 0.7, F(interaction)_3, 47_ = 1, p(interaction) = 0.4, Fig. 3B). Consistent with the analysis on arrival times, high-dimensional behavioural analysis, performed on the subset of metrics validated for their ability to detect STM (Fig. 2C, Table S2), showed no significant differences that passed multiple comparison corrections between DBS-ON and DBS-OFF conditions. Overall, these findings indicate that NAc-DBS does not significantly facilitate or interfere with spatial STM formation.

#### NAc-DBS effects on long-term memory retrieval

We next assessed the effect of NAc-DBS on the retrieval of previously encoded LTM. We found that animals during DBS-ON sessions retain the ability to find the previous day ROI (S1) earlier than the current ROI (S2) at T1 of S2 (P(*t*_S1ROI_ < *t*_S2ROI_) = 0.611, H_0_: *t*_closest_ < *t*_furthest_ rejected, Table S1). The behavioural analysis, performed on the subset of metrics validated for their ability to detect LTM (Fig. 2D, Table S3), showed no difference between DBS-ON and OFF conditions (Fig. 3D). These results demonstrate no positive nor negative interference of NAc-DBS on the retrieval of previously acquired memories.

#### NAc-DBS effects on long-term memory encoding

We then investigated the effect of NAc-DBS on LTM encoding by assessing target navigation on T1 of S3. This is, we assessed, in the absence of stimulation (S3), the retrieval of memories encoded the previous day (S2) in the presence (DBS-ON) or absence (DBS-OFF) of NAc stimulation. First, the probability to first reach the target confirmed the ability of the animals to find the ROI location encoded in S2 before than the current ROI location of S3 (P(*t*_S2ROI_ < *t*_S3ROI_) = 0.611, H_0_: *t*_closest_ < *t*_furthest_ rejected, Table S1). Importantly, animals that were stimulated in S2 (DBS-ON) showed more efficient navigation towards the S2 ROI on S3 than non-stimulated ones (Fig. 3E, Table S4), suggesting improved memory formation by NAc-DBS. Specifically, stimulated animals covered less distance and explored a smaller area to find the target (*d, d (center), A, A(center)*, Fig. 3E). Their movements were also more ballistic towards the target (*α min. Dist., α min. Dist. (ELE)*, Fig. 3E). These results remained significant after multiple comparison correction using the Benjamini-Hochberg method, controlling for a FDR of 0.1 (Table S4). Notably, with six significant results, the expected number of false positives is 0.6 i.e. between zero and one test. Thus, the conclusions drawn from the collective evidence remain robust following correction.

Overall, these findings suggest that DBS in the NAc may exert a favourable influence on the encoding phase of memory formation, without significantly disrupting either the retrieval of memories during the stimulation period or affecting other measured behavioural parameters to a notable extent.

#### Effect of NAc-DBS on reinforcing, anxiolytic, or motor effects

Given the extensive dopaminergic afferents in the NAc [20], we investigated potential reinforcing or anxiolytic effects of DBS.

For the reinforcing effect, we used a conditioned place preference (CPP) task by applying the NAc-DBS while the animal was in one of the 3 chambers of the apparatus (see Supplementary Information). In this paradigm, stimulation in the initially less-preferred chamber is expected to increase preference for that chamber if the stimulation is rewarding. A one-tailed paired t-test comparing DBS-ON and DBS-OFF conditions showed a marginally significant difference (t = 2.04, df = 5, p = 0.05, 95% CI [−0.03, 0.27]; Fig. 3F), suggesting a possible, though limited, reinforcing effect.

Anxiety evaluation was conducted using the elevated plus maze (EPM, see Supplementary Information) and revealed no significant effect of DBS. The proportion of time animals spent exploring the open arms of the maze did not significantly differ between DBS-ON and -OFF conditions (two-tailed paired t-test, t = 0.35, df = 6, p = 0.7, Fig. 3G).

Potential stimulation-induced motor alterations are unlikely, as demonstrated by the absence of significant differences in the animals’ navigation speed (*v*, *v(center)*) when exploring the arena in the first trial of S2 (LTM retrieval test) while the stimulation is ON vs. OFF. The tortuosity of trajectories (*α(t) persistence*) also did not change (Fig. 3D). We argue that a direct effect on motor function should be evident in all DBS-ON trials, which, as we indicated above, was not the case.

At the end of the behavioural experiments, the position of the DBS electrodes in the NAc was verified (Fig. 3H). Furthermore, there was no excessive tissue reaction to the implanted electrode, as assessed by astroglial staining.

### Changes in brain activity induced by NAc-DBS

**Fig. 4:**
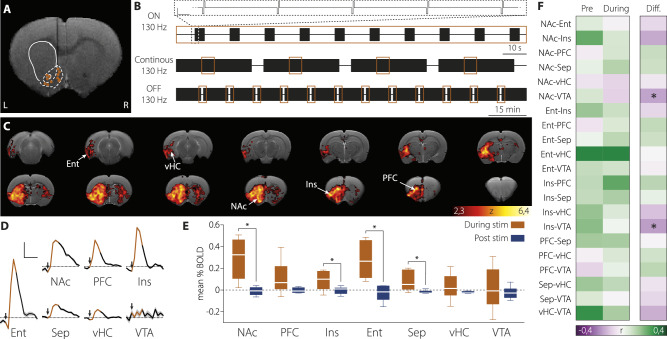
Acute and chronic effects of NAc-DBS on brain-wide activity. **A** High resolution anatomical image (T2-weighted) showing the location of the stimulation electrode in each animal (while lines representing the striatal area, with the core and shell parts of the NAc delimited with dotted lines). The vertical thin dark shadow is the artefact produced by the carbon-fibre DBS electrode. **B** Schematic representation of the stimulation protocols used. Top: acute DBS-ON paradigm (8 s ON, 22 s OFF). Scale 1 ms, 300 μA. Middle: continuous DBS paradigm. fMRI data (10 min) is recorded during continuous DBS stimulation. Bottom: acute DBS-OFF paradigm. After a period of sustained DBS (10 min) the stimulus is switched off and the fMRI response is recorded. Orange squares represent acquisition windows. **C** Thresholded cluster-corrected functional maps (z > 2.33, cluster p = 0.05) of the animals stimulated with the acute DBS-ON paradigm overlaid on a T2-weighted anatomical image. n = 12. **D** BOLD signal time courses evoked by the acute DBS-ON in selected brain regions, represented as % change vs. baseline. Black arrows mark the start of the stimulation and orange tracing the duration of the stimulus. Scale 10 s, 0.5%, n = 12. **E** BOLD signal changes in the acute DBS-OFF paradigm. Orange bars: mean BOLD signal in the last 22 s of the 10 min continuous stimulation. Bue bars: mean BOLD signal in the first 22 s after stimulation cessation. Median and min-to-max whiskers represented, n = 6, *p < 0.05. **F** Pearson correlation between the mean BOLD signals in selected brain regions during continuous DBS vs. resting-state pre-stimulation (Pre) conditions. Right panels show the difference between both conditions n = 4, *p(Benjamini-Hochberg corrected) < 0.05. NAc nucleus accumbens, PFC prefrontal cortex, Ins insular cortex, Ent entorhinal cortex, Sep septum, vHC ventral hippocampus, VTA ventral tegmental area.

Using MRI-compatible stimulation electrodes and recording fMRI signals during the application of the DBS protocol, we investigated changes in brain activity induced by the stimulation (Fig. 4). Acute stimulation of the NAc in blocks of 8 s (Fig. 1B, see ‘Materials and Methods’) evoked robust blood oxygenation level dependent (BOLD) signal responses and enabled us to map brain regions afferent and efferent to the stimulated area, including *en passant* axons. Major evoked responses by acute stimulation were found in the NAc and other striatal regions, the prefrontal, orbitofrontal, insular and entorhinal cortices, the septum and the ventral hippocampus (Fig. 4C, D). The observed activations were predominantly ipsilateral, with only limited activation in discrete regions of the contralateral striatum.

To investigate the effect of DBS during a chronic application, as performed in the behavioural experiments above, we substituted acute by continuous stimulation and recorded fMRI signals in 10 min blocks. We then computed functional connectivity as the correlations between the average BOLD signals recorded in the regions identified in the previous experiment (Fig. 4D). We compared functional connectivity before and during DBS application (Fig. 4F). Significant changes in correlation between the measured brain regions were induced by the continuous stimulation (one-way MANOVA, F_21, 6_ = 4.17, p = 0.04, Wilks’ lambda = 0.064, Fig. 4F). Pairwise comparisons for the 21 pairs of brain regions revealed statistically significant differences between DBS-ON and OFF conditions between the ventral tegmental area (VTA) and the NAc (F_1, 26_ = 12.42, p(Benjamini-Hochberg corrected) = 0.02, FDR correction for the 21 comparison tests), and between the insular cortex and the VTA (F_1, 26_ = 20.85, p(Benjamini-Hochberg corrected) = 0.002, FDR correction for the 21 comparison tests), decreasing in both cases with NAc-DBS.

The direct effect of continuous electrical stimulation at high frequency on neuronal activity is not well understood. Arguments in favour of a neuronal inactivation in response to a prolonged stimulation can be found in the literature [12, 13, 21, 22]. To investigate this hypothesis, we acquired fMRI signals during the last 22 s of a 10-min stimulation period and for the next 22 s after the end of stimulation (Fig. 4B). If neuronal response to DBS retains a certain level of activity in the continuous protocol, the BOLD signal is expected to decrease after stimulus termination. Conversely, if neuronal response is completely habituated to stimulation, no change in BOLD signal level is expected after the stimulus is switched off. Alternatively, if continued stimulation actively inhibits the target area, rebound activity would be expected due to disinhibition upon cessation of stimulation. We found that the mean BOLD signal significantly decreased upon DBS cessation in the NAc (two-tailed paired t-test, t = 3.66, df = 5, p = 0.01), entorhinal cortex (two-tailed paired t-test, t = 3.32, df = 5, p = 0.02), insular cortex (two-tailed paired t-test, t = 3.54, df = 5, p = 0.02) and septum (two-tailed paired t-test, t = 2.93, df = 5, p = 0.03), with no measurable rebound activity in any structure (Fig. 4E). These findings reveal that, under the current experimental conditions and throughout the 10-min duration of the behavioural task, continuous DBS induced a sustained activity increase across several of the targeted structures, including the NAc.

## Discussion

We provide evidence supporting a positive effect of DBS in the NAc on long-term memory formation. Specifically, we demonstrated that DBS can facilitate the encoding of new memories without affecting the retrieval of existing memories or interfering with ongoing short-term memory processes. This efficacy, combined with the absence of significant appetitive or anxiolytic side effects, underscores the considerable potential of NAc-DBS for clinical application. Our research also highlights the importance of employing high-dimensional behavioural analysis, which provided a more nuanced understanding of DBS facilitating mechanistic interpretations. Importantly, most of the NAc-DBS-driven effects on memory performance reported here would have otherwise gone unnoticed using standard ‘time-to-target’ behavioural measurements. Lastly, by combining NAc-DBS and simultaneous whole-brain measurements with fMRI, we provide evidence for sustained vs. putatively phasic effects of chronic stimulation on distal brain areas.

### Learning in the newly developed navigation task

The procedure developed allowed effective memory encoding of each daily location, with performance typically characterised by a monotonic decrease in latency to find the ROI across trials in each session (Fig. 2B). The asymptotic performance in trials 2–4 reflects the effectiveness of the memory update. High-dimensional analysis showed that better memory formation was indicated by more effective navigation of the maze, covering a shorter distance and exploring a smaller area of the maze before finding the target, with more frequent and faster forays to the central zone being made from the wall position nearest to the target ROI, with directed and ballistic trajectories.

In each session, the animals first recalled the memory of the ROI’s previous location and subsequently updated their memory by encoding its current location within the session. A binary classifier confirmed the presence of LTM in our task, aligning with approaches commonly used in traditional memory paradigms. In contrast, our high-dimensional analysis provides a more detailed and quantitative assessment of memory performance. The same set of high-dimensional features indicated STM updating with the new location of the ROI within each session (between T1 and T4) and demonstrated LTM between sessions (at T1 of S2 or S3, compare Fig. 2A, C). To assess the impact of experimental manipulations on memory, we focused on features that show significant first-order effects, as these are most likely to capture meaningful changes. From a Bayesian perspective, only metrics sensitive to baseline memory processes can reliably detect manipulation-induced effects on STM or LTM. While a gradient boosted trees model could help identify the most predictive features in a potentially less biased manner, the limited dataset currently constrains the application of such models.

Overall, the behavioural task and analysis provide a robust framework for evaluating the effects of interventions on distinct facets of memory formation. This is achieved within a continuous task repetition paradigm, which helps mitigate the impact of small group sizes that are often challenging to increase due to the complexity of some animal preparations. By maximising the data obtained from each subject, the framework also minimises the number of animals required for a study. Additionally, using light as a mild discomfort stimulus represents a significant improvement over more invasive or stressful methods, such as food or water deprivation, or the swim-or-sink paradigm used in water maze tasks. Notably, because light is not a strongly aversive stimulus, we argue that its effectiveness as a discomfort stimulus diminishes across sessions, gradually shifting toward a role more akin to cueing stimulus.

### Dissecting NAc-DBS effects using the navigation task

The NAc is implicated in the reward system and is integrated within the memory network [10, 23, 24]. We show that NAc-DBS increases LTM performance, an effect it produces by facilitating the codification of new information rather than facilitating the recall of previously stored information, supporting a critical online role of this structure for memory enhancement [6]. This differentiation is crucial for interpreting the effects of DBS and planning its potential therapeutic use.

Furthermore, the absence of significant changes in the high-dimensional behavioural space analysed during retrieval dismisses the notion of unspecific enhancement of other cognitive or executive functions by NAc-DBS. Moreover, the counterbalanced experimental design also rules out cumulative DBS effects that could confound task performance along sessions. Finally, direct DBS-induced LTP is unlikely to underlie the observed effects, as electrically induced LTP is typically associated with memory occlusion rather than enhancement [25].

In a broader context, we propose that the facilitation of memory encoding by NAc-DBS may promote a more flexible memory system, enabling more efficient cycles of encoding, recall, updating, and consolidation of updated information. In a preliminary human study [6], an improvement in episodic memory formation has been reported in a cohort of OCD patients, and one patient with anorexia nervosa, receiving 130 Hz electrical stimulation in the NAc [6]. While not establishing a causal link between NAc-DBS-induced memory enhancement and clinical improvement in OCD or anorexia nervosa, we argue that increased cognitive flexibility may contribute to overall cognitive function and potentially synergize with the therapeutic effects of NAc-DBS in these conditions, which are marked by cognitive rigidity [3]. Moreover, this enhanced memory updating process may help explain why the clinical benefits of NAc-DBS typically emerge progressively over time rather than immediately [2].

Finally, despite the dense reciprocal connectivity with the VTA and the importance of the dopaminergic system in appetitive behaviour and anxiety [10, 26–28], we observed only a minor effect on appetitive behaviour, with no observable effect on anxiety. These results facilitate the interpretation of the behavioural changes attributed to memory effects.

The histological analysis revealed that the electrode tips (Figs. 3H, 4A) were distributed across both the core and shell regions of the NAc. However, considering the reported stimulation radius of ~2 mm for protocols with parameters similar to those used in this study [19], functional differentiation between these subregions is not feasible in our study. Therefore, we pooled core and shell DBS location for the analysis.

### Brain-wide interactions of NAc-DBS

Acute stimulation in the NAc at 130 Hz engaged a network of memory-related brain regions extending from the NAc to the septum, insular, prefrontal and entorhinal cortices and the ventral hippocampus, in agreement with previous fMRI studies [29]. However, stimulation is delivered continuously in most DBS protocols, including ours. In those conditions, brain activations in response to stimulation habituated to some degree, but retained supra-baseline levels in the NAc, septum, insular cortex and entorhinal cortex. Sustained activation of this network may explain why a reversible memory effect of NAc-DBS is observed in patients even after long periods of DBS [6]. Furthermore, continuous DBS decreased the functional connectivity between the VTA and the NAc, and between the VTA and the insular cortex.

Previous studies suggested that NAc-DBS primarily induces antidromic activation of corticostriatal fibres and inhibits local cell bodies within the targeted nucleus [19, 30]. However, our fMRI results showed sustained activation within the NAc itself, as well as in the septum, insular cortex, and entorhinal cortex during stimulation. This BOLD response rises above baseline during DBS and returns to pre-stimulation levels afterward, indicating metabolic engagement of the stimulated tissue. These findings do not exclude the involvement of afferent or *en passant* axons—well-documented in the literature—but suggest that, under our experimental conditions, DBS produces a net local activation in the NAc. This activation likely contributes to downstream (orthodromic) functional connectivity alongside antidromic effects during stimulation.

Sustained BOLD activity in the NAc by DBS may involve continuous dopamine release from VTA axons, which could act as a saliency signal enhancing memory encoding [10]. Additionally, direct activation of NAc efferent GABAergic projections to the VTA [31, 32]—originating from D1-expressing medium spiny neurons and targeting GABAergic interneurons—has been shown to induce a disinhibited state in the VTA, further increasing dopaminergic activity [32]. This may, in turn, promote dopamine release in the hippocampus and other memory-related structures [10, 32]. Supporting this interpretation, we observed a shift from a positive to a negative correlation between BOLD signals in the VTA and NAc (Fig. 4F). Furthermore, the small but statistically significant effect of NAc-DBS in the CPP task (Fig. 3F) also suggests increased dopamine release. This disinhibited state would enhance the responsiveness of the VTA to inputs coming from different brain regions. A larger variety of inputs driving VTA activity may also account for the change in BOLD signal correlations between the VTA and the NAc and insular cortex (Fig. 4F). Our results support a long-lasting reorganisation of the functional balance in the mesocorticolimbic system, resulting in sustained enhanced activity in regions critical for memory formation.

When conducting fMRI experiments, the use of anaesthesia necessitates caution in directly interpreting brain connectivity results to explain cognitive processes in awake animals. Although this limitation is widely acknowledged, it is also important to highlight some advantages of using urethane as an anaesthetic agent. Urethane has been shown to induce brain states more akin to physiological sleep than to coma-like states [33] and, unlike other commonly used anaesthetics such as isoflurane, minimally impacts blood pressure and blood flow [34]. This characteristic is particularly relevant to our study, as fluctuations in these parameters could significantly influence the acquired BOLD signals. Additionally, urethane’s capacity to maintain a stable depth of anaesthesia over extended periods [35] proved especially beneficial for our experimental paradigm, which involved analysing the evolution of whole-brain interactions under the cumulative effects of DBS treatment over time. Future studies implementing an awake animal preparation for DBS-fMRI will be instrumental in assessing the extent to which these findings translate to the unanesthetized state.

Finally, we acknowledge that the use of only male rats limits the generalisability of our findings across sexes. While female patients have received NAc-DBS in clinical settings with no reported differences in treatment efficacy [2], future research should systematically compare male and female responses to NAc-DBS. Additionally, although pre-selecting the most exploratory animals helps reduce the number of subjects required, it may also restrict the applicability of the findings to broader populations. In future experiments, it will also be interesting to map the effects of NAc-DBS to discriminate functional differences between the core and shell subregions. This will likely require dedicated protocols with lower stimulation intensity or pulse duration [19] to gain anatomical specificity.

Overall, the combination of an animal model enabling the dissection of various stages of memory formation with a high-dimensional behavioural analysis has led us to conclude that NAc-DBS selectively facilitates the updating or encoding of new information into memory without exerting positive or negative effects on recall and without significantly impacting other cognitive or executive functions. DBS-fMRI experiments further demonstrated that chronic stimulation induces sustained activation of the mesocorticolimbic system, prominently involving the dopaminergic system, which we suggest emulates a dopaminergic novelty signal that enhances memory encoding. We further propose that the therapeutic benefit of NAc-DBS in various psychiatric conditions may result from enhancing cognitive flexibility by promoting an updated memory base.

## Supplementary information

Supplementary information

## Data Availability

All data will be available at DIGITAL.CSIC repository.
