# Supplementary material for: NAc-DBS selectively enhances memory updating without effect on retrieval
Source: Neuropsychopharmacology. 2025 May 27;50(9):1420–9. doi: 10.1038/s41386-025-02132-0 (PMC12259920; doi:10.1038/s41386-025-02132-0)
Supplement: Supplementary file 1 — Supplementary information [file 41386_2025_2132_MOESM1_ESM.docx]

**SUPPLEMENTARY INFORMATION**

**Supplementary methods**

***Experimental design***

The first experiment was aimed to validate the newly developed behavioural task (n = 6). The second experiment was devoted to perform DBS of the NAc (n = 4). From an initial group of 18 rats we selected for each group those exhibiting greater exploratory behaviour to proceed with the experiments. This selection was made over the first two days of habituation by excluding animals that spent most of their time close to the walls of the arena, as subsequent memory analysis would rely on characterizing the exploratory behaviour of the animals. The integrity of the implants for DBS during the whole duration of the extensive behavioural training and tests conditioned the permanence of the animals in the study. These two experiments comprised a total of 95 behavioural experimental tests, including DBS sessions of three linked sessions (S1-3, see *The navigation task* section) in a within-subject manner of active (current on) and sham (current off) stimulation, which were statistically compared and increased the statistical power of the comparison. The two conditions were counterbalanced for each animal.

The third behavioural experiment involved the evaluation of possible anxiolytic or appetitive effects of the NAc-DBS treatment (n = 7). In CPP experiments all rats underwent DBS in the less preferred chamber for each rat (see *Conditioned place preference*). EPM was performed by randomly interleaving DBS-ON and -OFF periods during the exposure of the animals to the maze.

The fourth experiment was a fMRI investigation of brain networks modulated by DBS. The number of repetitions of each stimulation paradigm used is specified below. We utilised 12 male Sprague-Dawley rats, purchased and housed under similar conditions.

***The navigation task***

The task takes place within a circular arena measuring 180 cm in diameter, featuring four entering/exit boxes situated at the North, East, South, and West positions. The doors of these entering boxes are operated by pneumatic pistons. The apparatus is housed in a temperature-controlled room set at around 21 ºC, provided with visual cues on the walls outside the maze. The apparatus is cleaned with distilled water and 70 % ethanol between trials to avoid any odor cue. A recording camera is positioned above the maze (Flea3, Teledyne FLIR, USA), along with a dimmable light source. The recorded image is analysed and processed in real-time to control the maze based on the tracking of the animal's behaviour. All components of the apparatus are integrated into an Arduino UNO board (Arduino LCC) and orchestrated using the Bonsai visual reactive programming software^36^, synchronised with online animal tracking.

In the task, upon entering the arena through one of the boxes, the animal navigates under high illumination conditions (approximately 400 lux) to locate a user-defined virtual (invisible for the rat) platform. Once the rat locates the virtual platform, the light switches off and the entering door reopens, allowing the animal to return to the box. In this dry version of the Morris water maze, the natural aversion of rats to intense light serves as both an aversive stimulus and a cue to signal when the “platform” has been reached, motivating the animals to actively seek for it (Fig. 1A). In regular experiments, the virtual platform has a diameter of 12 cm and can manifest in any of the 16 predetermined locations (see Fig.1B).

One week prior to commencing training, animals undergo 7 days of handling, spending 10 minutes daily within the experimentation room under comfortable light conditions. Subsequently, they undergo a 5-day habituation protocol. During the first two days, the animals freely explore the arena without illumination by being placed directly inside the arena. Over the next three days, they enter the arena from randomly selected entering boxes, intensively illuminated, while the arena remains dark. Once the door opens, the animals explore the arena for 10 minutes, repeating this task twice daily with a 20-minute delay. The initial two days of habituation help identify animals that spend more time in the central area, ensuring a group with highly exploratory individuals (Fig. 1D).

In the initial training phase, the animals learn the contingencies of the task (a specific location in the large arena switches off the light and opens an escape pathway to conclude the task). This phase comprises three stages over three consecutive days, starting with large virtual platforms and progressively reducing their size. The first stage starts with a one-quadrant size in the centre of the arena, followed by half a quadrant, and concludes with the final size of the “platform”. Animals are considered trained at this point. Figure 1D provides a schematic representation of the complete schedule.

A specific feature of the design allows dissociation of memory formation and memory recall and adapts the delay-matched-to-place protocol^14,17^ previously developed for the water maze. The animals are extensively trained in this task for 15 sessions which are, importantly, structured in successive groups of 3 sessions called S1-S3 (Fig 1C). An experimental session in one session consists of 4 trials (T1-4) of up to 10 min in which the virtual platform remains in the same position (Fig. 1C). The location of the virtual platform changes the next day, and it is pseudo randomly moved between the 16 locations across successive sessions. Thus, on T1, the animals may remember the location of the virtual platform of the previous session one day earlier; however, across trials T1 to T4, they will encode a new location and so form a new memory. This new location may be remembered on T1 of the next day. Accordingly, during the first 90 sec of T1, the virtual platform is inactive to allow precise monitoring of the searching behaviour of the animals. By having successive groups of 3 sessions together, DBS could be applied on S2 to examine its impact on recall of memory formed on S1, its impact on short-term memory during S2, and its impact on the formation of long-term memory measured on T1 of S3 (Fig. 1C).

Memory function is estimated by extracting different measures from the tracking of the animals (see *Behavioural data preprocessing* section) related to the search for the previous day's region of interest (ROI, enlarged version of the virtual platform with a diameter of 24 cm, LTM) and how it adapts to the new virtual platform location (STM). Non-experimental test experiments with no manipulation and maintaining the “platform” location between days, occur between triads of experiments to keep the animals engaged in the task.

***Behavioural data processing***

The Bonsai visual reactive programming software^36^ was employed for online tracking to operate the apparatus based on the animal's location in the arena. The received camera image underwent processing with an HSV threshold to generate a binary image, facilitating the detection of when the animal reached specified locations.

For data analysis, precise tracking of the animals (nose, left and right ears, shoulders, back, and the base of the tail, Fig.1B) was extracted using the freely accessible software DeepLabCut^37,38^ (see *Tracking analysis*). Due to visual differences between groups (wired head implants in the DBS experiment), two distinct models were trained for data analysis—one for the non-stimulated animals and another for the DBS and control conditions. The diameter of the ROI for detecting exploration in the subsequent analysis was increased 2 times in order to prevent slight tracking imprecisions and to compensate for the distance between the shoulders (the main body part used in the analysis) and the silhouette of the animal (used to operate the apparatus).

In the navigation task, the evidence of STM is derived from the exploration of the ROI in the 4 consecutive trials of a given day. For each trial, we meticulously analysed the navigation patterns of the animals towards the target. A comprehensive set of 20 metrics designed to delineate various aspects of navigation was extracted, which fell into 5 main categories: directionality towards target, wall navigation strategies, ballisticness, spatial navigation, and speed (see Table 1). The variation of these metrics between trials 1 (T1) and 4 (T4) concerning the current ROI location was used to evaluate STM. Subsequently, LTM is quantified by statistically comparing the variation of previously described behavioural metrics in the T1 of a given day concerning the current ROI and the previous day’s ROI. The median statistic is employed to penalise outliers and one-tailed confidence intervals with confidence level 95 % were then used to determine significance (see *Tracking analysis*).

The original set of 20 metrics was primarily designed to evaluate the presence of STM and LTM (first-order effects). The selection of second-order metrics, used to assess differences in memory performance between DBS and control groups, was pre-determined based on their ability to measure first-order effects (see *Behavioural features analysis*). Additionally, a decision model was applied to detect LTM. If a rat exhibits LTM, it will tend to explore the ROI from the previous day first during T1, rather than the new, unknown ROI. This defines a null model for the absence of LTM: a binary classifier predicting that the rat will first locate the target closest to its entry point into the arena. If this null model performs worse than the proportion of rats finding the previous day's ROI first, the null model is rejected, and LTM is confirmed (see *Long-term memory evaluation*).

All calculations for the offline analysis were performed in Python and other standard statistical procedures were computed in SPSS (IBM Corp. Released 2022. IBM SPSS Statistics for Windows, Version 29.0) and GraphPad Prism (GraphPad Software, Version 8).

***Tracking analysis***

DeepLabCut software^37,38^ was used to extract the precise position of the selected body parts of the animals across each frame of the videos. Each condition (wired and non-wired animals) involved preparing two sets of 16 videos for training. For each set, 40 frames of each video were utilised to label desired body parts (nose, left and right ears, shoulders, back, and the base of the tail, Fig.1B). Subsequently, 95% of the labelled frames were used for training the network.

A ResNet-101-based neural network^39,40^ was employed with default parameters for 1.3 x 10^6^ training iterations and three consecutive training sessions. The results were validated through a single shuffle, yielding a test error of 3.95 pixels and a train error of 3.5 pixels for the control condition. In the DBS condition, the test error was 5.17 pixels, and the train error was 3.9 pixels. The image size used was 1552 x 1552 pixels. Subsequently, these networks were applied to analyse the remaining recorded videos for each respective group.

***Long-term memory evaluation***

For LTM detection and regarding the probability of a rat in finding the target ROI or any other ROI in the arena, we defined a null model (lack of LTM): a binary classifier predicting that a rat first finds the target (either ROI or previous day’s ROI) whose location is closer to the rat’s entering point to the arena:

$$X=\left( x_{1}, \ldots, x_{n} \right), x_{i}= \{1, d\left( rat, p.ROI \right)<d\left( rat, ROI \right), 0, d\left( rat, p.ROI \right)>d\left( rat, ROI \right),$$

The null model is then evaluated against the actual outcome (finding first the previous day’s ROI or the ROI at each T1) across rats and days:

$$Y=\left( y_{1}, \ldots, y_{n} \right), y_{i} = \{1, if rat finds preROI, 0, if rat finds ROI.$$

Lack of LTM suggests that this null model should outperform a blind classifier that simply predicts the majority class (without using any data). To compare both classifiers, the F_1_ score is used, and if the blind classifier performs better than the null model with 95 % confidence, then the null model is rejected, and it is concluded that LTM is detected.

***Behavioural features analysis***

In the quantification of STM and LTM by comparing the evolution of the described behavioural features (Table 1) across trials, variation is expressed as the metric’s ratio (*r*). The median statistic is employed to minimize the influence of outliers. Due to differences in the distributions of each metric and the lack of prior information, separate analyses were conducted using non-parametric methods. The metrics evaluated both the presence of STM and LTM as first-order effects, while variations in these memory functions between the Control and DBS groups were assessed as second-order effects. To address potential inflation of the FDR from multiple comparisons, the Benjamini-Hochberg correction was applied. The FDR was controlled at 0.05 for first-order effects and 0.1 for second-order effects, considering the limited sample size and the greater complexity of the second-order analyses.

To compute confidence intervals for STM and LTM evaluation (first-order effect), comparisons are made using paired observations (e.g. a rat in T1 vs the same rat in T4). Exact, distribution-free confidence intervals for *r* are utilised^41^. A paired contrast involves comparing two sets of data, *X* and *Y*, where each set has an equal number of observations, denoted by *n_1_=n_2_=n*, and each element in *X* is uniquely associated with a corresponding element in *Y*. This type of comparison is appropriate for comparing the results of different trials for rats that have undergone the same treatment. For a given metric evaluated in a certain treatment and the control group, with values *x_i_* and *y_i_* respectively, the variable of interest is:

$$r_{i}= \frac{y_{i}}{x_{i}}-1, i=1, \ldots, n.$$

[ 1 ]

Thus, *r_i_* > 0 (*r_i_* < 0) implies an increase (decrease) in the metric in set Y compared to X with respect to the control group. As observations are paired, the empirical distribution of *r* can be computed and, following Hahn & Meeker^41^, the exact, distribution-free, one-sided confidence lower and upper bound with confidence level 1-𝛼, for the quantile *x_p_* are given by the order statistics *x_(l)_* and *x_(u)_*, where:

$$l=n- q_{1- \alpha;n;1-p^{'}} u= q_{1- \alpha;n;p^{'}}+1,$$

[ 2 ]

being $q_{\alpha;n;p}$ the quantile 𝛼 of a binomial distribution *B(n, p)*. The exact, equally-tailed confidence interval with confidence 1-𝛼 is given by

$$\left[ x_{(l)}, x_{(u)} \right],$$

[ 3 ]

Where *l* and *u* are obtained from [2] using a significance level 𝛼/2.

When evaluating DBS-ON *vs*. -OFF, the goal is to assess variations in memory (second-order effect). In these cases, pairing individual observations is not feasible. Thus, confidence intervals are computed using a bootstrap method, where pairing occurs at the rat level (block-pairing). Let *X* and *Y* represent the control and treatment groups' observation sets, which contain subsets associated with each rat:

$$X= \bigcup_{k=1}^{S}X_{k}, Y= \bigcup_{k=1}^{S}Y_{k},$$

[ 4 ]

where *S* is the number of rats. Note that the following condition must be met:

$$N_{x}= \sum_{k=1}^{S} n_{X}^{(k)}, N_{Y}= \sum_{k=1}^{S} n_{Y}^{(k)},$$

[ 5 ]

where *N_X_* and *N_Y_* represent the total number of observations for the control and treatment groups, and $n_{X}^{(k)}$ and $n_{Y}^{(k)}$ are the number of observations associated with rat *k*.

The procedure involves the following steps:

1. Conduct *R =* 10^4^ resamples with replacement on the paired subsets labelled by the rat (*X_k_, Y_k_*).

2. For each subset *X_k_* and *Y_k_*, resample their contents *R_B_* = 10^3^ times.

3. Calculate the expected values of *X_k_* and *Y_k_*.

4. Compute the variable of interest using the same method as in the paired case with equation [1], using $\underline{X}_{k}$ and $\underline{Y}_{k}$ instead of *x_i_* and *y_i_*.

5. Obtain the confidence interval using the standard percentile method with the total computed resamples $R_{T}=R \cdot R_{B}= {10}^{7}$.

Steps 1 and 3 ensure that the conditions for a paired comparison are met, while step 2 accounts for the uncertainty in the expected values.

The original 20 metrics were designed to evaluate the presence of STM and LTM as first-order effects. However, changes in STM and LTM induced by DBS represent second-order effects, building upon the basic presence of memory. From a Bayesian perspective, the likelihood of a metric reliably measuring second-order effects depends on its ability to detect first-order effects. Consequently, to compare memory differences between DBS-ON and DBS-OFF conditions, we included only metrics that were statistically significant in detecting memory presence. Metrics unable to capture the first-order effect, even with a larger sample size, are unlikely to reliably measure second-order effects and were excluded from further analysis. This approach focuses on validated metrics with greater statistical power, reducing the number of tests required and minimizing FDR inflation from multiple comparisons.

***Conditioned place preference***

To investigate the potential appetitive or aversive motivational effects of the stimulation, a CPP protocol was employed. In this paradigm, conditioned preference is assessed by measuring the time spent in the chamber paired with the stimulus before and after conditioning. The protocol spanned three days as follows:

On day 1 animals were allowed to freely explore the apparatus for 15 minutes without any stimulation. The second day the animal was confined to the chamber where it had spent less time in the previous session (non-preferred chamber), and DBS was applied for 30 minutes. On the last day, the animals were once again given the opportunity to freely explore the apparatus without stimulation for 15 minutes.

The proportion of time spent in the conditioned chamber was then quantified to assess the place preference.

***Elevated plus maze***

The EPM serves as a test to assess anxiety levels in experimental animals. The model is based on the natural aversion of rodents to open spaces and their preference for staying in enclosed ones. The test employs an elevated, plus-shaped apparatus with two open arms and two enclosed arms. Anxiety reduction is indicated by an increase in the proportion of time spent in the open arms (time in open arms/total time in open and closed arms).

The experiment comprised a 10-minute duration trial, split into two periods of DBS-ON and two periods of DBS-OFF, each lasting 2 minutes and 30 seconds. These periods were intercalated, randomly assigned, and counterbalanced. The comparison of the time spent by the animals in the open arms versus the closed arms between ON and OFF periods served as an index for evaluating the potential anxiolytic effect of NAc-DBS.

***Chronic implantation surgery***

Following the completion of the behavioural training, the animals underwent bilateral implantation of bipolar custom-made electrodes in the NAc. The rats were anaesthetised using isoflurane (induction at 5 %, maintenance at 1.5-2.5 % in oxygen) and received local analgesia through a lidocaine injection (50 mg/ml). Positioned in a stereotaxic frame (Narishige, Japan), the animals' temperature was maintained at 37 ºC using a heat blanket (Cibertec, Spain), and heart and breath rates were monitored to adjust the isoflurane concentration (MoseOx Plus, Starr Life Sciences, USA). Ophthalmic gel was applied to keep the animal's eyes hydrated throughout the procedure.

Electrodes were constructed by twisting two teflon-coated platinum-iridium filaments (A-M Systems, USA), resulting in 200 μm in diameter electrodes, with an impedance of 80-100 kΩ. Two of these electrodes were soldered to a Lemo connector (Fig. S1A, 1S series, Lemo, Switzerland) to connect to the pulse generator and current source (STG-2004, Multi-Channel Systems, Germany) through a custom-made cable (Fig. S1A). Electrodes were targeted at both NAc through trephines drilled in the skull. The target coordinates were anteroposterior (AP) 1.5 mm from bregma, mediolateral (ML) 0.8 mm, and dorsoventral (DV) 6.5 mm from dura (Fig. 3H), following the Paxinos and Watson atlas^42^. To ensure implant durability, two screws were affixed to the skull, and the implant secured in place with dental cement (Superbond, Sun Medical, Japan).

Towards the end of the procedure, animals received a buprenorphine injection (3 μg/kg). For the subsequent three days, or until signs of discomfort ceased, they were provided with pellets infused with buprenorphine (0.003 mg/ml). After three days, the animals were rehoused in pairs and allowed to recover for a minimum of 7 days before any behavioural testing continued.

***MRI-compatible surgery***

Functional MRI experiments were performed under urethane anaesthesia (1.3 g/kg, i.p.), with oxygen supplied during surgical procedures as previously described. To minimise artefacts during electrical stimulation inside the scanner, glass-coated carbon-fibre bipolar electrodes developed in the laboratory were employed as previously described^43,44^. Individual carbon fibres with a diameter of 7 μm (Goodfellow Cambridge Limited, UK) were inserted into bundles within a theta-shaped glass capillary (World Precision Instruments) previously pulled from 7 cm long pipettes. This configuration resulted in a tip of 200 μm and was adjusted to achieve an electrical impedance of 40-65 kΩ. A regular wire with a gold pin connector was attached to the pipette, connected to the carbon fibres using silver conductive epoxy resin (RS Components, UK), and isolated with clear epoxy resin. Subsequently, the tip was bent in a flame to a 90º angle to minimise implant height, allowing for close proximity of the MRI array coil and the animal's brain (Fig. S1B). This preparation has been shown to provide robust fMRI results in rats^45^.

These carbon-fibre electrodes were inserted into the NAc at coordinates 1.2 mm AP, 0.8 mm ML, and 6.6 mm DV (Fig. 4A). The electrode was firmly secured in place with MRI-compatible dental cement (Heraeus Medical, Wehrheim, Germany), and the animal was then transferred to the scanner.

***fMRI experiments and data analysis***

For the fMRI experiments, the previously prepared urethane-anaesthetised animals were positioned in a custom-made animal holder with adjustable bite and ear bars, and then placed on the magnet bed. A constant supply of 0.8 l/min O_2_ was provided, and the temperature was maintained between 37 and 37.5ºC using a water heat pad. Temperature, heart rate, and SpO2 were monitored as during surgeries. The experiments were conducted in a horizontal 7 T scanner with a 30 cm diameter bore (Biospec 70/30, Bruker Medical, Ettlingen, Germany). A 1H rat brain receive-only phased-array coil with an integrated combiner and preamplifier, and no-tune/no-match, was utilised along with the actively detuned transmit-only resonator (Bruker BioSpin MRI GmbH, Germany).

Acquisition involved 15 coronal slices using a gradient echo-echo planar image (GE-EPI) sequence with the following parameters: field of view (FOV) 25 x 25 mm, slice thickness 1 mm, matrix 96 x 96, segments 1, flip angle (FA) 60º, time echo (TE) 15 ms, time repetition (TR) 2 s. For the acquisition of the 130 Hz trains, a total of 150 volumes were acquired over a 5-minute duration, and 3 repetitions of this protocol were acquired per animal for averaging purposes. In the continuous stimulation paradigm, 4 repetitions of 150 volumes were acquired in the middle of the 30-minute stimulation sequence (see Fig. 4B). Similar acquisitions were conducted before any stimulation to establish a baseline record of activity in the absence of stimulation.

T_2_-weighted anatomical images were obtained using a rapid acquisition relaxation enhanced sequence (RARE) with parameters: FOV 25 x 25 mm, 15 slices, slice thickness 1 mm, matrix 192 x 192, TE_eff_ 56 ms, TR 2 s, RARE factor 8.

fMRI data were analysed offline using custom software developed in Python, which included the Advanced Normalization Tools Ecosystem (ANTs, https://github.com/ANTsX), FSL Software (https://fsl.fmrib.ox.ac.uk/fsl/fslwiki/FSL), and Analysis of Functional Neuroimage (AFNI, https://afni.nimh.nih.gov/).

The images were augmented 10 times, and anatomical ones were utilised to create a common template for brain extraction mask generation. Anatomical images underwent bias field correction and were registered to the custom template for brain extraction. The preprocessing of functional images included motion correction (McFlirt), brain extraction with registration to the template, intensity normalisation, and temporal (high-pass 0.01 Hz) and spatial filtering (FWHM with a kernel ~1.5x voxel size).

Activation maps were processed using FEAT (FSL’s FMRI Expert Analysis Tool). A time series representing each of the stimulation trains was convolved with a double-gamma hemodynamic response function shifted forward in time by 2 seconds, which was then used for the general linear model analysis. Time-series statistical analysis was conducted using FILM (FMRIB's Improved Linear Model) with local autocorrelation correction^46^. Higher-level analysis was performed using a fixed-effects model for averaging between runs, and a mixed-effects model utilising FLAME (FMRIB’s Local Analysis of Mixed Effects) with automatic outlier detection^47-49^ for subjects averaging. z (Gaussianised T/F) statistical images were thresholded using clusters determined by z > 2.33 and a corrected cluster significance of p = 0.05^50^.

The BOLD time series from regions of interest were extracted by registering the subjects to an anatomical atlas^51^. This registration facilitated the calculation of the mean BOLD response in those regions triggered by each stimulation train as a percentage relative to a peristimulus baseline of 8s. Additionally, the Pearson correlation between different structures was computed using the BOLD time series from the registered ROIs in both resting and stimulation conditions.

***Histology***

To verify the accurate placement of implanted elements and assess potential excessive tissue damage, immunohistochemical analyses were conducted.

Following the completion of experiments, the animals underwent transcardial perfusion with a phosphate saline buffer at 37 ºC, followed by 4% paraformaldehyde at 4 ºC. Subsequently, the fixed brains were extracted from the skulls and immersed in paraformaldehyde at room temperature overnight. Coronal slices of 100 μm thickness were obtained using a vibratome (Leica Biosystems, Germany).

For labelling glial fibrillary acidic protein, a mouse monoclonal antibody (Sigma Aldrich, USA) was utilised, followed by a secondary anti-mouse Alexa Fluor 488-conjugated antibody (Thermo Fisher Scientific, USA). Slices were further stained with 4’,6-diamino-2-phenylidole. Fluorescence microscope images were captured (Leica Microsystems, Germany) to visualise the location of the cannulas and the glial reaction. Image analysis was performed using the ImageJ software^52^.

**Supplementary references**

1. Lopes G, Bonacchi N, Frazão J, Neto JP, Atallah BV, Soares S, et al. (2015) Bonsai: an event-based framework for processing and controlling data streams. Front Neuroinform 9:7. doi:10.3389/fninf.2015.00007
2. Mathis A, Mamidanna P, Cury KM, Abe T, Murthy VN, Mathis MW, et al. (2018) DeepLabCut: markerless pose estimation of user-defined body parts with deep learning. Nat Neurosci 21:1281–1289. doi:10.1038/s41593-018-0209-y
3. Nath T, Mathis A, Chen AC, Patel A, Bethge M, Mathis MW (2019) Using DeepLabCut for 3D markerless pose estimation across species and behaviors. Nat Protoc 14:2152–2176. doi:10.1038/s41596-019-0176-0
4. Insafutdinov E, Pishchulin L, Andres B, Andriluka M, Schiele B (2016) DeeperCut: A Deeper, Stronger, and Faster Multi-person Pose Estimation Model. Computer Vision – ECCV 2016 34–50. doi:10.1007/978-3-319-46466-4_3
5. He K, Zhang X, Ren S, Sun J (2016) Deep residual learning for image recognition. 2016 IEEE Conference on Computer Vision and Pattern Recognition (CVPR). IEEE. doi:10.1109/cvpr.2016.90
6. Hahn GJ, Meeker WQ (1991) Statistical Intervals: A Guide for Practitioners. Wiley-Interscience. doi:10.1002/9780470316771
7. Paxinos G, Watson C (2006) The Rat Brain in Stereotaxic Coordinates: Hard Cover Edition. Elsevier. ISBN: 9780080475158
8. Pérez-Cervera L, Caramés JM, Fernández-Mollá LM, Moreno A, Fernández B, Pérez-Montoyo E, et al. (2018) Mapping Functional Connectivity in the Rodent Brain Using Electric-Stimulation fMRI. Methods Mol Biol 1718:117–134. doi:10.1007/978-1-4939-7531-0_8
9. Pacheco-Torres J, Moreno A, Fernandez B, Pérez-Cervera L, Carames JM, Fernandez-Molla LM, Perez-Montoyo E, Martinez-Sanchez M, Moratal D, Canals S (2018) in: Handbook of Behavioural Neuroscience (D. Manahan-Vaughan, Ed), vol. 28, chap. 24. Elsevier, London.
10. Moreno A, Morris RGM, Canals S (2016) Frequency-Dependent Gating of Hippocampal-Neocortical Interactions. Cereb Cortex 26(5):2105-2114. doi:10.1093/cercor/bhv033
11. Woolrich MW, Jbabdi S, Patenaude B, Chappell M, Makni S, Behrens T, et al. (2009) Bayesian analysis of neuroimaging data in FSL. Neuroimage 45:S173–86. doi:10.1016/j.neuroimage.2008.10.055
12. Woolrich MW, Ripley BD, Brady M, Smith SM (2001) Temporal autocorrelation in univariate linear modeling of FMRI data. Neuroimage 14:1370–1386. doi:10.1006/nimg.2001.0931
13. Beckmann CF, Jenkinson M, Smith SM (2003) General multilevel linear modeling for group analysis in FMRI. Neuroimage 20:1052–1063. doi:10.1016/S1053-8119(03)00435-X
14. Woolrich MW, Behrens TEJ, Beckmann CF, Jenkinson M, Smith SM (2004) Multilevel linear modelling for FMRI group analysis using Bayesian inference. Neuroimage 21:1732–1747. doi:10.1016/j.neuroimage.2003.12.023
15. Worsley KJ (2001) Statistical analysis of activation images. Oxford University Press. doi:10.1093/acprof:oso/9780192630711.003.0014
16. Barrière DA, Magalhães R, Novais A, Marques P, Selingue E, Geffroy F, et al. (2019) The SIGMA rat brain templates and atlases for multimodal MRI data analysis and visualization. Nat Commun 10:1–13. doi:10.1038/s41467-019-13575-7
17. Schindelin J, Arganda-Carreras I, Frise E, Kaynig V, Longair M, Pietzsch T, et al. (2012) Fiji: an open-source platform for biological-image analysis. Nat Methods 9:676–682. doi:10.1038/nmeth.2019


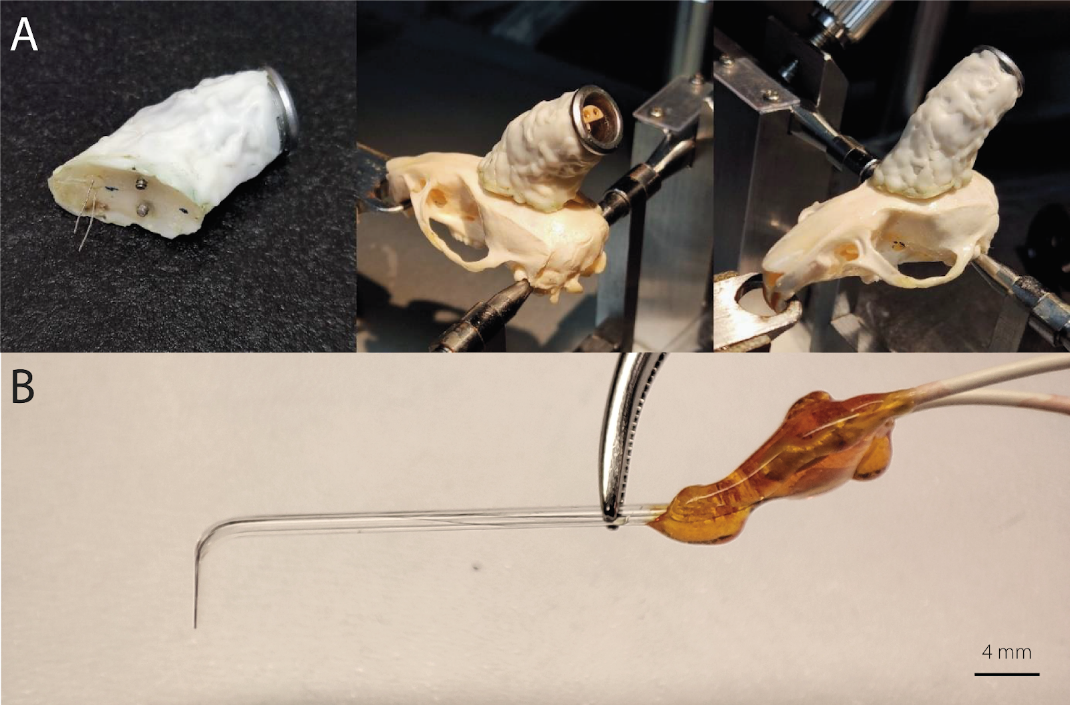


**Fig. S1. DBS implants. (A)** Behavioural chronic implant with bipolar bilateral Pt-Ir electrodes and its location over a rat skull. **(B)** Borosilicate-carbon fibre bipolar MRI-compatible electrode.
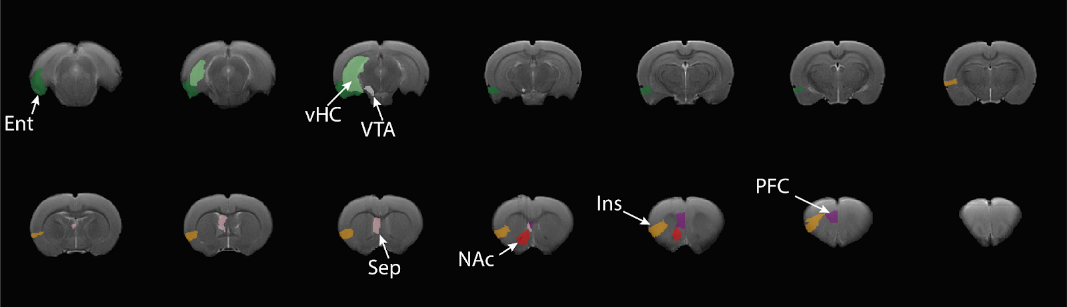


**Fig. S2. ROIs for mean BOLD extraction.** NAc: nucleus accumbens (red), PFC: prefrontal cortex (purple), Ins: insular cortex (orange), Ent: entorhinal cortex (dark green), Sep: septum (pink), vHC: ventral hippocampus (light green), VTA: ventral tegmental area (white).

| Group | n | Animals | P(t_preROI_ < t_ROI_) | F1_null_ - F1_blind_ | H_0_: t_closest_ < t_furthest_ |
| --- | --- | --- | --- | --- | --- |
| 1^st^ exp.:  Memory task | 60 | 6 | 0.63 ([0.51, 0.75]) | -0.16 ([-0.31, -0.05]) | Rejected |
| 2^nd^ exp.:  DBS-OFF | 19 | 4 | 0.68 ([0.47, 0.87]) | -0.17 ([-0.43, 0.01]) | Rejected |
| 2^nd^ exp.:  DBS-ON | 8 | 4 | 0.61 ([0.31, 0.89]) | -0.04 ([-0.67, 0.14]) | Rejected |
| 2^nd^ exp.:  postDBS | 8 | 4 | 0.61 ([0.31, 0.89]) | -0.37 ([-0.86, -0.1]) | Rejected |

**Table S1. LTM evaluation.** Experiments (group), observations (n), number of animals and metrics for arrival times used to evaluate LTM. P(*t*_preROI_ < *t*_ROI_) is the ratio of sessions finding before the ROI in the previous session (preROI) than the current ROI. This behaviour was not solely due to proximity to the starting position (F1_null_ - F1_blind_, H_0_: *t*_closest_ < *t*_furthest_; see Long-term memory evaluation). The postDBS group refers to animals receiving NAc-DBS on S2, with LTM assessed on T1 of S3.

| Metric | H1 (T4 vs T1) | p | q (BH) |
| --- | --- | --- | --- |
| *α(t) persistence ** | *Greater* | *0.030* | *0.046* |
| *Visits to target / d(center) ** | *Greater* | *0.004* | *0.006* |
| *Velocity ** | *Greater* | *<0.001* | *<0.001* |
| Velocity (center) | Greater | 0.055 | 0.068 |
| Area / d (center) | Greater | 0.542 | 0.542 |
| Area / t (center) | Greater | 0.457 | 0.481 |
| *Time ** | *Lesser* | *<0.001* | *<0.001* |
| *Wall probability ** | *Lesser* | *<0.001* | *<0.001* |
| *Distance ** | *Lesser* | *<0.001* | *<0.001* |
| *Distance (center) ** | *Lesser* | *<0.001* | *<0.001* |
| *Area ** | *Lesser* | *<0.001* | *<0.001* |
| *Area (center) ** | *Lesser* | *<0.001* | *<0.001* |
| *Distance to target (ELE) ** | *Lesser* | *<0.001* | *<0.001* |
| Recurrence time | Lesser | - | - |
| Recurrence time (center) | Lesser | - | - |
| *β ** | *Lesser* | *<0.001* | *<0.001* |
| β (center) | Lesser | 0.062 | 0.073 |
| *α until min distance ** | *Lesser* | *0.001* | *0.002* |
| α until min distance (ELE) | Lesser | 0.071 | 0.079 |
| Exploration count | Lesser | 0.055 | 0.068 |
| Dx to target (wall) | Lesser | 0.046 | 0.066 |
| *Dl to target (wall) ** | *Lesser* | *0.002* | *0.004* |

**Table S2. Selection of STM metrics based on effect detection in the memory evaluation group.** The q-values represent p-values adjusted for multiple comparisons, controlling the false discovery rate (FDR) using the Benjamini-Hochberg method. Metrics with q < FDR = 0.05 are in italic and marked with an asterisk (*). A hyphen indicates insufficient data (n < 2).

| Metric | H1 (preROI vs ROI) | p | q (BH) |
| --- | --- | --- | --- |
| *α(t) persistence ** | *Greater* | *0.005* | *0.010* |
| Visits to target / d(center) | Greater | 1.000 | 1.000 |
| Velocity | Greater | 0.390 | 0.429 |
| Velocity (center) | Greater | 0.057 | 0.082 |
| *Area / d (center) ** | *Greater* | *0.002* | *0.010* |
| Area / t (center) | Greater | 0.063 | 0.082 |
| *Time ** | *Lesser* | *0.003* | *0.010* |
| Wall probability | Lesser | 0.667 | 0.699 |
| *Distance ** | *Lesser* | *0.005* | *0.010* |
| *Distance (center) ** | *Lesser* | *0.005* | *0.010* |
| *Area ** | *Lesser* | *0.005* | *0.010* |
| *Area (center) ** | *Lesser* | *0.005* | *0.010* |
| *Distance to target (ELE) ** | *Lesser* | *0.001* | *0.005* |
| Recurrence time | Lesser | 0.062 | 0.082 |
| Recurrence time (center) | Lesser | 0.062 | 0.082 |
| β | Lesser | 0.390 | 0.429 |
| β (center) | Lesser | 0.080 | 0.098 |
| *α until min distance ** | *Lesser* | *<0.001* | *<0.001* |
| *α until min distance (ELE) ** | *Lesser* | *<0.001* | *<0.001* |
| Exploration count | Lesser | 0.054 | 0.082 |
| *Dx to target (wall) ** | *Lesser* | *<0.001* | *<0.001* |
| *Dl to target (wall) ** | *Lesser* | *0.003* | *0.010* |

**Table S3. Selection of LTM metrics based on effect detection in the first-order spatial memory analysis.** The q-values represent p-values adjusted for multiple comparisons, controlling the false discovery rate (FDR) using the Benjamini-Hochberg method. Metrics with q < FDR = 0.05 are in italic and marked with an asterisk (*).

| Metric | H1(DBS vs Control) | p | q (all) | q (filtered) |
| --- | --- | --- | --- | --- |
| α(t) persistence | Greater | 0.370 | 0.529 | 0.444 |
| Visits to target / d(center) | Greater | 0.330 | 0.529 |  |
| Velocity | Greater | 0.211 | 0.384 |  |
| Velocity (center) | Greater | 0.053 | 0.133 |  |
| Area / d (center) | Greater | 0.674 | 0.842 | 0.735 |
| Area / t (center) | Greater | 0.457 | 0.610 |  |
| Time | Lesser | 0.094 | 0.188 | 0.141 |
| Wall probability | Lesser | 0.951 | 0.951 |  |
| *Distance ** | *Lesser* | *0.048* | *0.133* | *0.096* |
| *Distance (center) ** | *Lesser* | *0.045* | *0.133* | *0.096* |
| *Area ** | *Lesser* | *0.034* | *0.133* | *0.096* |
| *Area (center) ** | *Lesser* | *0.041* | *0.133* | *0.096* |
| Distance to target (ELE) | Lesser | 0.085 | 0.188 | 0.141 |
| Recurrence time | Lesser | - | - | - |
| Recurrence time (center) | Lesser | - | - | - |
| β | Lesser | 0.933 | 0.951 |  |
| β (center) | Lesser | 0.801 | 0.942 |  |
| *α until min distance ** | *Lesser* | *0.020* | *0.133* | *0.096* |
| *α until min distance (ELE) ** | *Lesser* | *0.020* | *0.133* | *0.096* |
| Exploration count | Lesser | 0.048 | 0.133 |  |
| Dx to target (wall) | Lesser | 0.890 | 0.951 | 0.890 |
| Dl to target (wall) | Lesser | 0.359 | 0.529 | 0.444 |

**Table S4. Effects of DBS on LTM encoding.** The q-values represent p-values adjusted for multiple comparisons, controlling the false discovery rate (FDR) using the Benjamini-Hochberg method. q (all) refers to calculations considering all metrics, while q (filtered) includes only metrics that detected LTM in the first-order spatial memory analysis (Table S2). Metrics with q (filtered) < FDR = 0.10 are in italic and marked with an asterisk (*). A hyphen indicates insufficient data (n < 2).
